# Accuracy and Prognosis Value of the Sequential Organ Failure Assessment Score Combined With C-Reactive Protein in Patients With Complicated Infective Endocarditis

**DOI:** 10.3389/fmed.2021.576970

**Published:** 2021-03-25

**Authors:** Yaowang Lin, Shaohong Dong, Jie Yuan, Danqing Yu, Weijie Bei, Ruimian Chen, Haiyan Qin

**Affiliations:** ^1^Department of Cardiology, Shenzhen Cardiovascular Minimally Invasive Medical Engineering Technology Research and Development Center, Shenzhen People's Hospital (The Second Clinical Medical College, Jinan University; The First Affiliated Hospital, Southern University of Science and Technology), Shenzhen, China; ^2^Department of Cardiology, Guangdong General Hospital, Guangdong Academy of Sciences, Guangzhou, China; ^3^Department of Health Management, Shenzhen People's Hospital (The Second Clinical Medical College, Jinan University; The First Affiliated Hospital, Southern University of Science and Technology), Shenzhen, China

**Keywords:** SOFA, C-reactive protein, complicated infective endocarditis, in-hospital death, long-time outcome 3

## Abstract

This study aimed to evaluate the accuracy and prognostic value of the sequential organ failure assessment (SOFA) score combined with C-reactive protein (CRP) in patients with complicated infective endocarditis (IE). A total of 246 consecutive patients with complicated IE were included in the multicentric prospective observational study. These patients were divided into four groups depending on the SOFA score and CRP optimal cutoff values (≥5 points and ≥17.6 mg/L, respectively), which were determined using the receiver operating characteristic analysis: low SOFA and low CRP (*n* = 83), low SOFA and high CRP (*n* = 87), high SOFA and low CRP (*n* = 25), and high SOFA and high CRP (*n* = 51). The primary endpoint was in-hospital death, and the secondary endpoint was long-time mortality, defined as subsequent readmission and 3-years mortality in the follow-up period. High SOFA score and high CRP were associated with approximately 29.410% (15/51) of higher incidence of in-hospital death with an area under the curve of 0.872. Multivariate analyses showed that age [odds ratio (OR) = 2.242, 1.142–4.401], neurological failure (Glasgow Coma Scale ≤ 12) (OR = 2.513, 1.041–4.224), *Staphylococcus aureus* (OR = 2.151, 1.252–4.513), SOFA ≥ 5 (OR = 9.320, 3.621–16.847), and surgical treatment (OR = 0.121, 0.031–0.342) were clinical predictors for in-hospital death. On following up for 12–36 months, SOFA ≥ 5 (*p* = 0.000) showed higher mortality. A high SOFA score combined with increased CRP levels is associated with in-hospital mortality. Also, SOFA score, but not CRP, predicts long-term mortality in complicated IE.

## Introduction

Infective endocarditis (IE) causes nearly 20% of in-hospital mortality, 17% of 30-days mortality, 30% of 1-year mortality, and up to 40% mortality at 5-years follow-up, posing a diagnostic and therapeutic challenge to clinicians (1, 2). Therefore, early identification of patients at high risk of death or complications is essential to improve the outcome of this disease. Research works have shown the sequential organ failure assessment (SOFA) score and C-reactive protein (CRP) to be effective prognostic tools in the management of sepsis, infections as well as patients with IE (3–5). However, studies regarding the combined effect of SOFA and CRP on predicting adverse outcomes in patients with complicated IE remain unknown.

This is the first study documenting the combined effect of the SOFA score and CRP in predicting outcomes among patients with complicated IE.

## Materials and Methods

### Patients Enrollment

**Figure 1:**
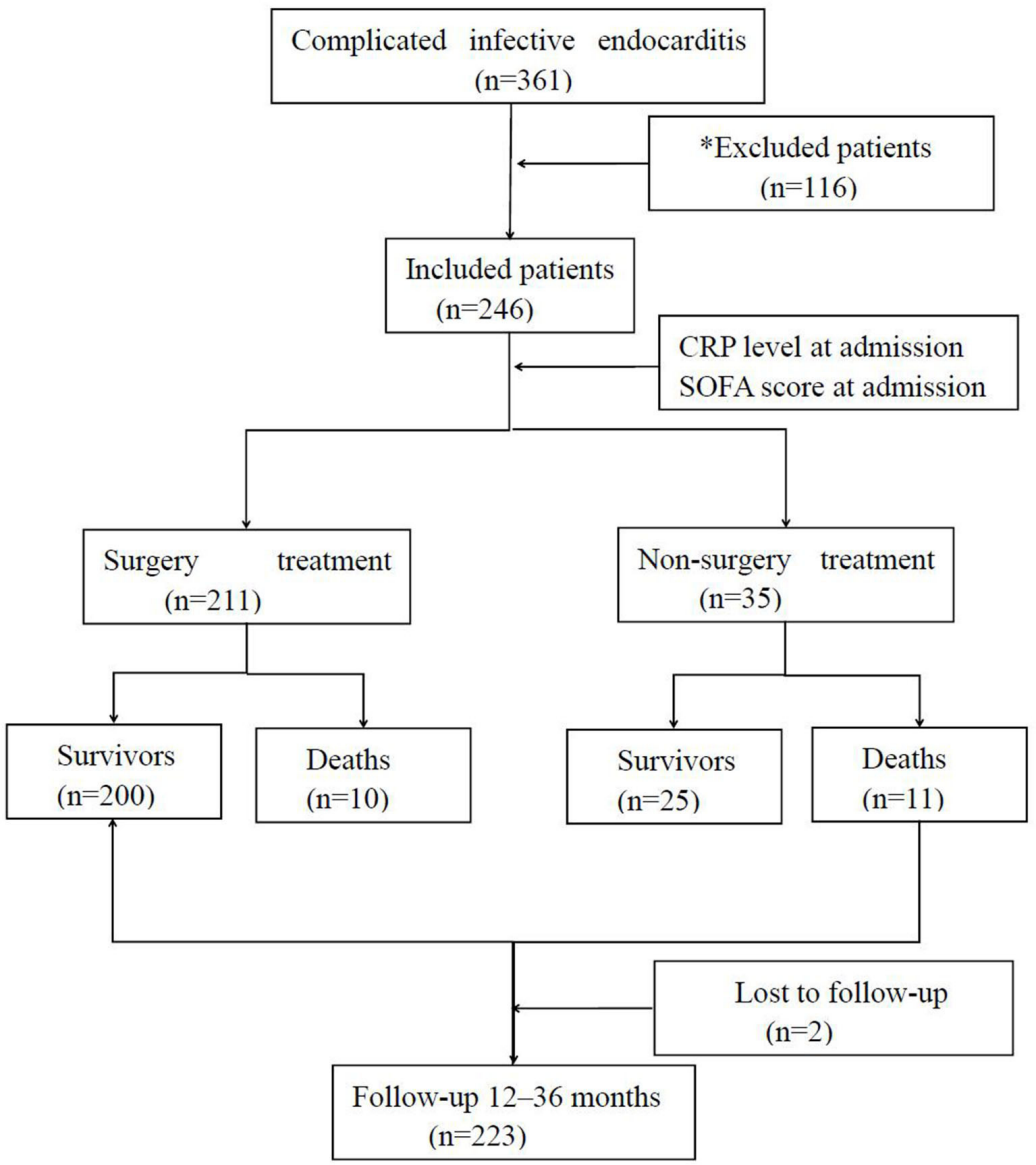
Flowchart of the statistical analysis. *Patients were excluded on having non-complicated infective endocarditis (*n* = 90), prior infective endocarditis (*n* = 10), age <18 years (*n* = 12), and others (*n* = 4).

A multicentric prospective observational study focusing on the impact of SOFA score and CRP level in evaluating the severity and the prognosis of complicated IE patients was conducted. The trial was conducted in six intensive care units in three big university-affiliated medical centers (Shenzhen People's Hospital, Longgang District People's Hospital of Shenzhen, and Guangdong General Hospital) in China. The minimal sample size of each group was calculated by the chi-square test used by PASS 15 software (6). A total of 246 patients definitively diagnosed with complicated IE were consecutively screened between 2015 and 2019. Based on modified Duke criteria (7), patients were confirmed to have either IE or complicated IE if they met one or more of the following criteria: (i) presence of congenital heart disease (CHD) including any type of cyanotic CHD or any type of CHD repaired with a prosthetic material up to 6 months after the procedure (8); (ii) neurological complication including ischemic stroke, intracerebral or subarachnoidal hemorrhage, brain abscess, meningitis, and toxic encephalopathy (9); (iii) paravalvular abscess identified by echocardiography; (iv) embolic complications including pulmonary, cerebral, or systemic embolism (10); or (v) heart failure (11). The exclusion criteria included no complicated IE, prior IE, or age younger than 18 years (Figure 1). Finally, the patients were divided into four groups depending on the respective optimal cutoff value. All patients gave written informed consent before their enrollment. The institutional review board at the Shenzhen People's Hospital approved the study protocol.

### Data Collection

Once the patients were enrolled, and serum was collected and sent for CRP analysis using an immunoturbidimetry assay with a range of 0–5 mg/L. Transthoracic echocardiography was performed, and SOFA scores were calculated within 24 h of diagnosis.

### Study Endpoints

In-hospital mortality was considered as the primary endpoint. Long-time mortality with follow-up was the secondary endpoint. Long-term mortality was defined as subsequent readmission and 3-years mortality in the follow-up period.

### Statistical Analysis

Statistical Package for the Social Sciences 22.0 was used for all statistical analyses in this study. The receiver operating characteristic curve analysis was used in search of the optimal cutoff value of SOFA or CRP for in-hospital mortality. Included patients were divided into four groups depending on the respective optimal cutoff value. Values were reported as mean ± standard deviations, quartile ranges, or counts (percentages). The continuous data results were compared using a Student's *t*-test, analysis of variance, or the Kruskal–Wallis test, whereas the chi-squared or Fisher's exact test was used to compare the distribution of categorical data. Univariable and multivariable logistic regression analyses were used to evaluate the adjusted odds ratio (OR) for in-hospital death. The Kaplan–Meier method was used to analyze long-time survival with 12–36 (24, 36) months of follow-up. A (*p* < 0.05) was considered statistically significant.

## Results

### Characteristics of the Patients

A total of 246 patients (178 males, mean age 44.00 ± 13.55 years) with complicated IE were included in this study. Patients were divided into four groups based on the SOFA score (≥5 points) and CRP level (≥17.6 mg/L) cutoff values: low SOFA and low CRP (*n* = 83), high SOFA and low CRP (*n* = 25), low SOFA and high CRP (*n* = 87), and high SOFA and high CRP (*n* = 51). Of 246 patients, 21 (8.54%) died during hospitalization. No statistically significant differences were found between the four groups with respect to demographic characteristics, risk factors, affected valve, echocardiographic findings, or clinical symptom. Patients with a high SOFA score and a high CRP level were associated with higher incidence of diabetes mellitus (9.8 vs. 3.45% vs. 0 vs. 0, *p* = 0.005), *Staphylococcus aureus* (27.45 vs. 9.20 vs. 12.0 vs. 2.41%, *p* = 0.001), stroke (27.45 vs. 16% vs. 8.05 vs. 1.2, *p* = 0.000), vegetation size ≥ 10 mm (56.86 vs. 47.13 vs. 44 vs. 29.63%, *p* = 0.014), in-hospital death (29.41 vs. 12 vs. 2.47 vs. 1.15%, *p* < 0.000), and long-time mortality (15.69 vs. 8 vs. 6.9 vs. 0%, *p* < 0.000). However, the rate of receiving surgery treatment was lower (68.63 vs. 88 vs. 88.51 vs. 95.06%, *p* = 0.000) (Table 1).

**Table 1:** Baseline clinical characteristics of patients according to SOFA and CRP.

**Characteristics**	**Low SOFA, Low CRP (*n* = 83)**	**Low SOFA, High CRP (*n* = 87)**	**High SOFA, Low CRP (*n* = 25)**	**High SOFA, High CRP (*n* = 51)**	***p-value***
Age (year)	41.86 ± 12.82	43.34 ± 13.93	47.88 ± 13.89	46.24 ± 13.52	0.126
Males, *n* (%)	64 (77.11)	56 (64.37)	18 (72.00)	40 (78.43)	0.781
AIE, *n* (%)	11 (13.25)	19 (21.84)	4 (16.00)	10 (19.61)	0.442
SIE, *n* (%)	72 (86.75)	68 (78.16)	21 (84.00)	41 (80.39)	0.512
Hypertension, *n* (%)	6 (7.22)	8 (9.20)	3 (12.0)	9 (17.65)	0.054
Diabetes mellitus, *n* (%)	0 (0)	3 (3.45)	0 (0)	5 (9.80)	*0.005*
Affected valve					
Aortic valve, *n* (%)	35 (42.17)	37 (42.53)	11 (44.0)	28 (54.90)	0.149
Mitral valve, *n* (%)	41 (49.40)	50 (57.47)	11 (44.0)	24 (47.06)	0.603
Triple vale, *n* (%)	7 (8.43)	13 (14.94)	3 (12.0)	6 (11.76)	0.612
Multiple valves, *n* (%)	8 (9.64)	9 (10.34)	2 (8.0)	4 (7.84)	0.745
Congenital heart disease, *n* (%)	14 (16.87)	8 (9.20)	3 (12.0)	6 (11.76)	0.416
Neurological failure (GCS ≤ 12), *n* (%)	6 (7.22)	7 (8.05)	2 (8.0)	3 (5.88)	0.788
Paravalvular abscess, *n* (%)	4 (4.82)	10 (11.49)	1 (4.0)	4 (7.84)	0.707
Stroke, *n* (%)	1 (1.20)	7 (8.05)	4 (16.0)	14 (27.45)	*0.000*
Heart failure, *n* (%)	41 (49.40)	43 (49.43)	12 (48.0)	31 (60.78)	0.238
NYHA III–IV, *n* (%)	35 (42.17)	38 (43.68)	8 (32.00)	28 (54.90)	0.273
LVEF (%)	63.50 ± 8.74	62.58 ± 9.78	67.12 ± 8.67	60.64 ± 9.38	*0.044*
Temperature, °C	38.8 ± 1.17	38.75 ± 0.57	38.95 ± 0.76	39.08 ± 0.72	0.922
Pathogen, n (%)					
*Staphylococcus aureus*	2 (2.41)	8 (9.20)	3 (12.0)	14 (27.45)	*0.001*
*Streptococci*	10 (12.05)	11 (12.64)	4 (16.0)	9 (17.65)	0.052
Healthcare-associated infection	2 (2.47)	2 (2.31)	1 (4.0)	3 (5.88)	0.425
WBC, × 10^9^/L	7.3 ± 2.6	9.4 ± 3.7	8.4 ± 3.7	11.4 ± 2.7	*0.041*
CRP, mg/L	7.10 ± 5.23	39.89 ± 27.29	8.98 ± 6.88	37.95 ± 17.18	0.000
SOFA score	3.08 (3, 4)	3.21 (3, 4)	5.68 (5, 7)	6.51 (5, 8)	0.000
ESR, mm/h	20.22 ± 20.51	54.30 ± 36.61	28.32 ± 27.45	36.67 ± 31.88	0.000
Vegetation size ≥10 mm, *n* (%)	24 (29.63)	41 (47.13)	11 (44.0)	29 (56.86)	*0.014*
Surgery treatment, *n* (%)	77 (95.06)	77 (88.51)	22 (88.0)	35 (68.63)	*0.000*
In-hospital deaths	2 (2.47)	1 (1.15)	3 (12.0)	15 (29.41)	*<0.000*
Longtime mortality	0 (0)	6 (6.90)	2 (8.0)	8 (15.69)	*<0.000*

*AIE, acute infective endocarditis; SIE, subacute infective endocarditis; NYHA, New York Heart Association; LVEF, left ventricular ejection fraction; WBC, white blood cell; CRP:C-reactive protein; ESR, erythrocyte sedimentation rate; SOFA, sequential organ failure assessment; GCS, Glasgow coma score. Italic values defined as reference value*.

### Predictive Value of the Sequential Organ Failure Assessment Score and the C-Reactive Protein Level for Adverse Outcomes

**Figure 2:**
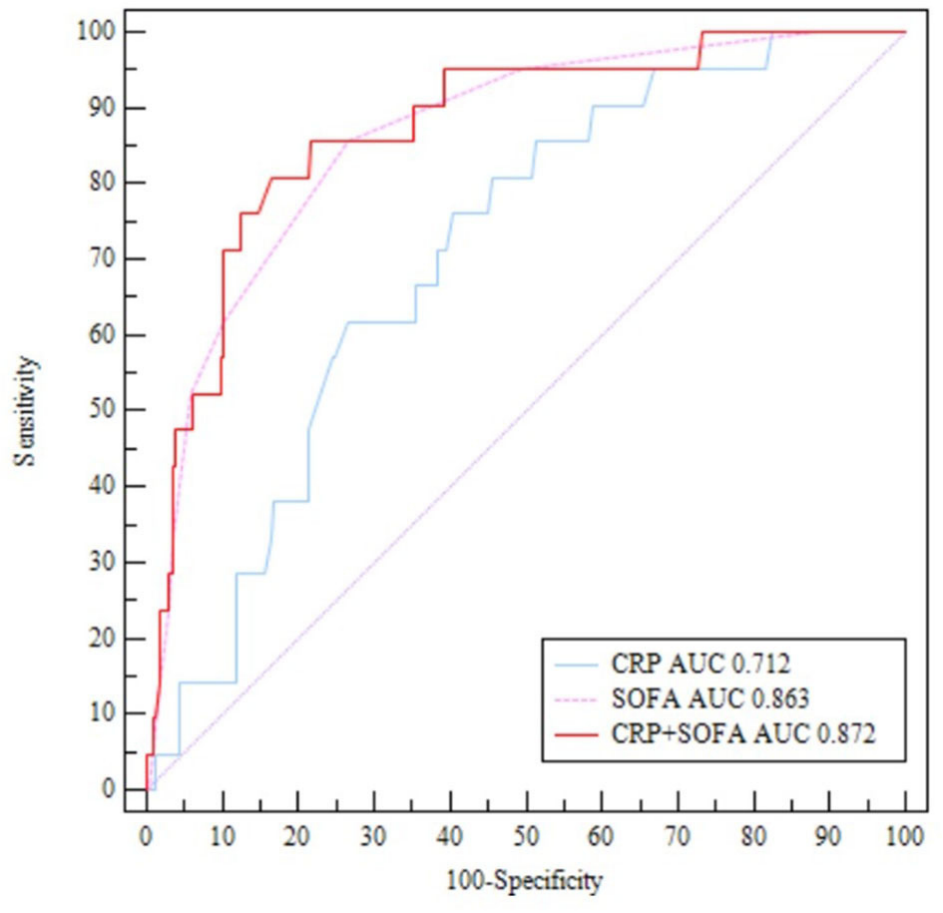
ROC curves for SOFA and CRP in predicting in-hospital death.

The receiver operating characteristic analysis revealed that SOFA score ≥ 5 was highly accurate in predicting the patient's in-hospital death [area under the curve (AUC) = 0.863, 95% confidence interval (CI), 0.814–0.904, *p* < 0.001] with a sensitivity of 85.71% and a specificity of 73.33%. CRP ≥ 17.6 mg/L was also accurate in predicting in-hospital death (AUC = 0.712, 95% CI, 0.651–0.768, *p* < 0.001) with a sensitivity of 85.71% and a specificity of 50.89%. The positive predictive values of SOFA score and CRP levels were 36.1 and 14.0%, respectively. The AUC of SOFA score combined with CRP in predicting patients' in-hospital death was 0.872 (95% CI, 0.825–0.912, *p* < 0.001) with a sensitivity of 80.95% and a specificity of 83.56% (Figure 2).

For in-hospital death, univariate analysis was used to identify predictive factors. Factors were related to a major risk for mortality including age (OR = 2.18, *p* = 0.002), hypertension (OR = 3.05, *p* = 0.048), neurological failure (Glasgow Coma Score ≤ 12; OR = 3.55, *p* = 0.041), *S. aureus* (OR = 2.45, *p* = 0.011), stroke (OR = 4.08, *p* = 0.009), heart failure (OR = 3.29, *p* = 0.025), CRP ≥ 17.6 mg/L (OR = 3.0, *p* = 0.038), and SOFA ≥ 5 (OR = 14.8, *p* < 0.000), whereas factor was related to survival was surgery treatment (OR = 0.11, *p* < 0.000). For in-hospital mortality, multivariate analysis revealed independent predictors such as age (OR = 2.242, 95% CI, 1.142–4.401, *p* = 0.015), Glasgow Coma Score ≤ 12 (OR = 2.513, 95% CI, 1.041–4.224, *p* = 0.012), *S. aureus* (OR = 2.151, 95% CI, 1.252–4.513, *p* = 0.020), surgery treatment (OR = 0.121, 95% CI, 0.031–0.342, *p* < 0.00), and SOFA ≥ 5 (OR = 9.320, 95% CI, 3.621–16.847, *p* = 0.001) (Table 2).

**Table 2:** Univariate and multivariate analyses of factors associated with in-hospital mortality.

**Characteristics**	**Survivors (*n* = 225)**	**All-cause death *(n* = 21)**	**Univariate HR (95% CI)**	***p-value***	**Multivariate HR (95% CI)**	***p-value***
^*^Age (year)	43.23 ± 13.19	54.77 ± 14.10	2.18 (0.66–4.52)	*0.002*	2.242 (1.142–4.401)	*0.015*
Males, *n* (%)	164 (72.89)	14 (66.67)	1.34 (0.52–3.49)	0.543		
AIE, *n* (%)	40 (17.78)	4 (19.05)	–	–		
SIE, *n* (%)	185 (82.22)	17 (80.95)	0.92 (0.29–2.88)	0.884		
Hypertension, *n* (%)	21 (9.33)	5 (23.81)	3.05 (1.01–9.12)	*0.048*		
Diabetes mellitus, *n* (%)	6 (2.67)	2 (9.52)	3.84 (0.73–20.36)	0.114		
Affected valve						
Aortic valve, *n* (%)	97 (43.11)	13 (61.90)	2.07 (0.83–5.20)	0.120		
Mitral valve, *n* (%)	117 (52.0)	9 (42.86)	0.91 (0.36–2.26)	0.831		
Triple vale, *n* (%)	28 (12.44)	1 (4.76)	0.34 (0.04–2.67)	0.308		
Multiple valves, *n* (%)	20 (8.89)	2 (9.52)	1.01 (0.4–2.56)	0.761		
Congenital heart disease, *n* (%)	28 (12.44)	3 (14.29)	1.16 (0.32–4.20)	0.820		
Neurological failure (GCS ≤ 12), *n* (%)	14 (6.22)	4 (19.05)	3.55 (1.05–11.97)	*0.041*	2.513 (1.041–4.224)	*0.012*
Paravalvular abscess, *n* (%)	17 (7.56)	2 (9.52)	1.28 (0.28–5.97)	0.752		
Stroke, *n* (%)	20 (8.89)	6 (28.57)	4.08 (1.42–11.6)	*0.009*		
Heart failure, *n* (%)	105 (46.67)	16 (76.19)	3.29 (1.16–9.28)	*0.025*		
NYHA III–IV, *n* (%)	96 (42.)	13 (61.90)	2.18 (0.87–5.48)	0.100		
#LVEF (%)	63.10 ± 9.21	60.33 ± 24	0.39 (0.14–1.11)	0.78		
WBC	7.3 ± 3.1	8.8 ± 2.7	1.18 (0.28–2.97)	0.702		
*Staphylococcus aureu*	23 (10.22)	4 (19.05)	2.45 (1.25–5.57)	*0.011*	2.151 (1.252–4.513)	*0.020*
*Streptococci*	31 (13.78)	3 (14.29)	1.05 (0.5–2.97)	0.841		
CRP <17.6 mg/L, *n* (%)	109 (48.66)	5 (23.81)	–	–		
CRP≥17.6 mg/L, *n* (%)	116 (51.56)	16 (76.19)	3.00 (1.07–8.49)	*0.038*		
^*^#ESR mm/h	35.96 ± 27.41	38.38 ± 23.73	1.69 (0.62–4.60)	0.309		
Vegetation size ≥10 mm, *n* (%)	92 (40.89)	13 (61.90)	2.31 (0.92–5.81)	0.074		
Surgery treatment, *n* (%)	200 (89.29)	10 (47.62)	0.11 (0.04–0.28)	*<0.000*	0.121 (0.031–0.342)	*<0.000*
Emergency, *n* (%)	30 (13.39)	1 (4.76)	–	–		
Urgent, *n* (%)	39 (17.41)	1 (4.76)	–	–		
Select, *n* (%)	131 (58.48)	6 (28.57)	–	–		
SOFA at admission, *n* (%)						
SOFA 0–4 points	166 (73.78)	3 (14.29)	–	–		
SOFA ≥5 points	59 (1.78)	18 (14.29)	14.88 (4.80–39.38)	*<0.000*	9.320 (3.621–16.847)	*0.001*

* Age cutoff was 56 years. #LVEF cutoff was 58%. *#ESR cutoff was 56 mm. AIE, acute infective endocarditis; SIE, subacute infective endocarditis; NYHA, New York Heart Association; LVEF, left ventricular ejection fraction; CRP, C-reactive protein; ESR, erythrocyte sedimentation rate; SOFA, sequential organ failure assessment; GCS, Glasgow coma score. Italic values defined as reference value.

### Long-Time Outcomes

**Figure 3:**
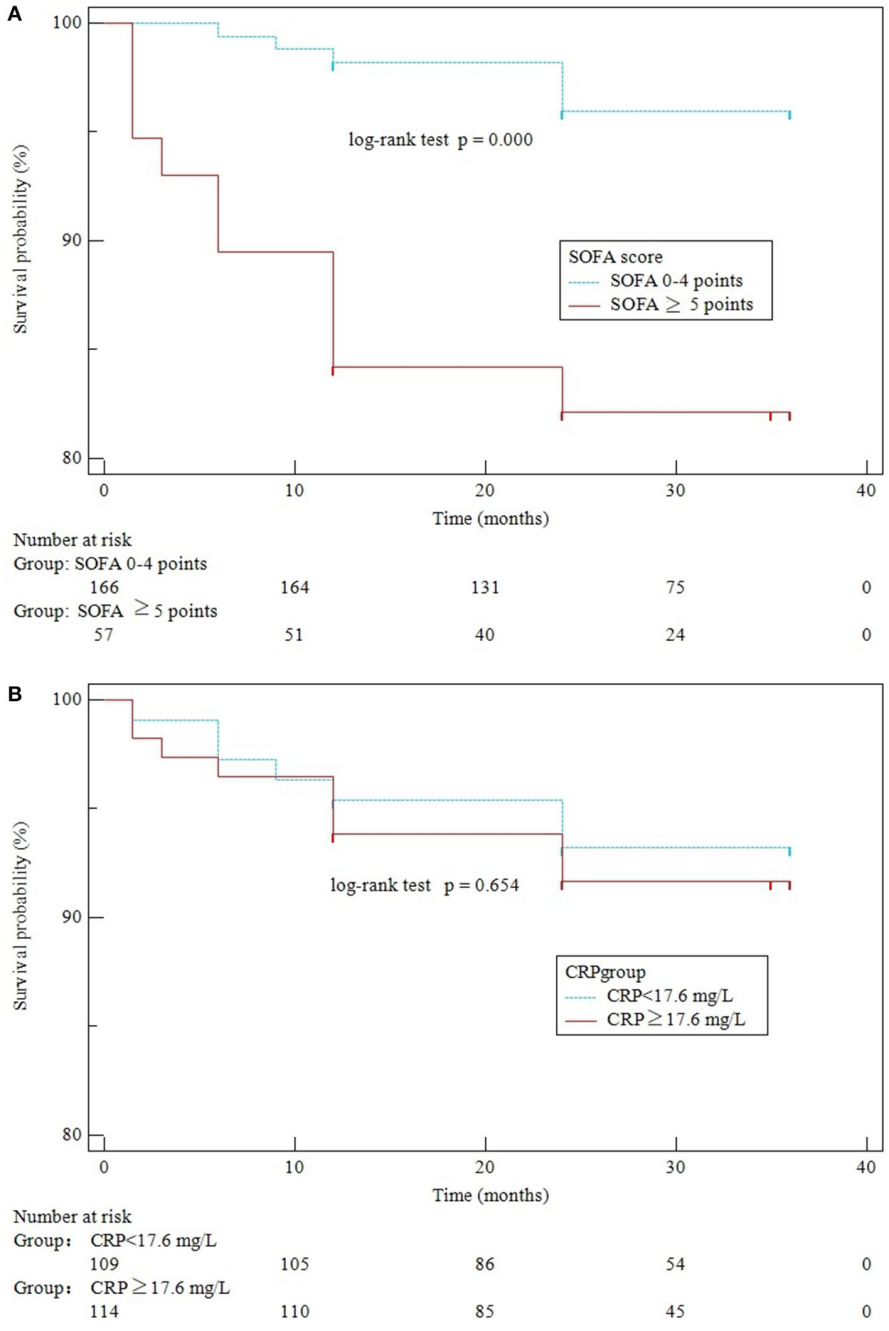
Kaplan–Meier curves of SOFA scores **(A)** and CRP levels **(B)** for survival over 3 years of follow-up.

Among the 225 patients after hospitalization, 2 (0.89%) patients were lost to follow-up. A total of 16 (7.17%) patients were dead within a follow-up time of 12–36 (24, 36) months. A lower cumulative rate of the long-term survivors with SOFA≥5 (log-rank test, *p* = 0.000) was demonstrated by the Kaplan–Meier analysis. However, we did not observe any significant difference in disease-free survival for CRP (log-rank test, *p* = 0.654) (Figure 3).

## Discussion

This study is the first clinical trial to evaluate the combinational effect of SOFA and CRP in predicting mortality in patients with complicated IE. The major findings are as follows: (i) strong predictive value of SOFA ≥ 5 combined with CRP ≥ 17.6 mg/L for in-hospital mortality; (ii) high SOFA score, but not CRP, is independently associated with long-time mortality.

This is the first study documenting the combined effect of the SOFA score and CRP in predicting outcomes among patients with complicated IE. IE is a severe disease, causing 15–30% of in-hospital mortality (12, 13). However, early diagnosis of some prognostic factors may help in decreasing the mortality rates. A SOFA score of 2 or more was confirmed to be valid means of identifying sepsis with suspected infection and was demonstrated to be a significant predictor of intensive care unit mortality (14). CRP is an acute-phase inflammatory serum protein that responds rapidly to infection and is highly accurate in predicting sepsis-suspected mortality in patients (5). Endocarditis with bacteremia leads to organ dysfunction and embolic complications in IE. Therefore, the SOFA score and the CRP level are determined to study the severity and prognosis of IE. The study by Kim et al. showed that higher SOFA [7(4–11) vs. 3(1–5), *p* < 0.001] was associated with poor outcomes in *S. aureus* bacteremia in IE (15). The data published by Asai et al. confirm that calculating the SOFA score could be a prognostic method for predicting in-hospital mortality in IE patients with a cutoff of 6 (AUC 0.915, sensitivity 76.9%, specificity 89.6%) (4). In our study, the SOFA score combined with the CRP level was considered a valuable factor in risk stratification (AUC = 0.872, sensitivity 80.95%, specificity 83.56%). The results of our study showed lower AUC and higher sensitivity when compared with those of Asai (that included coagulase-negative bacteremia patients without IE as a control group). A high SOFA score combined with increased CRP was significantly associated with in-hospital mortality with high sensitivity and specificity in this study.

The results from our study revealed that surgery treatment (OR = 0.121, *p* < 0.000) was a protective predictor, and *S. aureus* (OR = 2.151, *p* = 0.020) was an adverse predictor for the outcome during hospitalization in patients with complicated IE (16, 17). Patients with *S. aureus* infection are at the highest risk of death and need surgery in the active phase of the disease (18). Poor organ failure and more comorbidities among patients with high SOFA scores and high CRP levels might be associated with the low rate of receiving surgical procedures (68.63 vs. 88 vs. 88.51 vs. 95.06%, *p* = 0.000), likely attributing to the high death rate.

There is a high correlation of high CRP with a high SOFA score confirmed by the Spearman correlation test (analyze/correlate/bivariate) with r = 0.81 (*p* = 0.004). Therefore, the relation found of CRP with mortality was in fact indirect to the relation with SOFA score, and only SOFA ≥ 5 (OR = 9.320) was statistically significant in multivariate analysis.

As per the result of our study, high SOFA but not CRP is associated with high long-term mortality. CRP is an acute-phase inflammatory serum protein that is reactive to sepsis and can be suppressed through effective antimicrobial therapy or surgical treatment. However, cardiac or non-cardiac complications combined with patient characteristics are the main factors leading to a bad prognosis (19). The SOFA score was calculated for factors such as respiration, coagulation, liver function, circulatory systems, central nervous system, and renal function, which reflects the severity of organ failure and predicts underlying comorbidities (20).

However, there are a few limitations to this study. First, embolic complications are asymptomatic, therefore are not included in this study. Second, the SOFA score and CRP level should be calculated, recorded, and compared (i) before surgery, (ii) after surgery, and (iii) before discharge. Additionally, neurological complications after surgery were not included, which might contribute to postsurgical mortality. Finally, the sample size was small and only patients with complicated IE were included. However, this may not be applicable to all IE patients.

## Conclusion

This study concluded that the SOFA score combined with the CRP level is a valuable prognostic tool to evaluate complicated IE. SOFA ≥ 5 combined with CRP ≥ 17.6 mg/L was significantly associated with in-hospital mortality. Also SOFA score, but not CRP, predicts long-term mortality in complicated IE.

## Data Availability Statement

The raw data supporting the conclusions of this article will be made available by the authors, without undue reservation.

## Ethics Statement

The studies involving human participants were reviewed and approved by Shenzhen People's Hospital. The patients/participants provided their written informed consent to participate in this study. Written informed consent was obtained from the individual(s) for the publication of any potentially identifiable images or data included in this article.

## Author Contributions

YL and HQ collected, analyzed, and wrote this manuscript. JY, DY, RC, and WB assisted in the conduct of the study. SD was the principal investigator. All authors contributed to the article and approved the submitted version.

## Conflict of Interest

The authors declare that the research was conducted in the absence of any commercial or financial relationships that could be construed as a potential conflict of interest.
